# Neurotrophin Receptor p75NTR Regulates Immune Function of Plasmacytoid Dendritic Cells

**DOI:** 10.3389/fimmu.2017.00981

**Published:** 2017-08-17

**Authors:** Joanna Bandoła, Cornelia Richter, Martin Ryser, Arshad Jamal, Michelle P. Ashton, Malte von Bonin, Matthias Kuhn, Benjamin Dorschner, Dimitra Alexopoulou, Katrin Navratiel, Ingo Roeder, Andreas Dahl, Christian M. Hedrich, Ezio Bonifacio, Sebastian Brenner, Sebastian Thieme

**Affiliations:** ^1^Department of Pediatrics, University Clinic Dresden, Dresden, Germany; ^2^Department of Medical Laboratory Sciences, Imperial College of Business Studies, Lahore, Pakistan; ^3^DFG-Center for Regenerative Therapies Dresden, Cluster of Excellence, Technische Universitaet Dresden, Dresden, Germany; ^4^Medical Clinic I, University Clinic Dresden, Dresden, Germany; ^5^DKTK-German Cancer Consortium, Partner Site Dresden, University Clinic Dresden, Dresden, Germany; ^6^DKFZ-German Cancer Research Center, Heidelberg, Germany; ^7^Faculty of Medicine, Institute for Medical Informatics and Biometry, Technische Universitaet Dresden, Dresden, Germany; ^8^BIOTEChnology Center/DFG-Center for Regenerative Therapies Dresden, Cluster of Excellence, Technische Universitaet Dresden, Dresden, Germany

**Keywords:** plasmacytoid dendritic cells, p75 neurotrophin receptor, neurotrophin, TLR9, autoimmune diabetes, graft-versus-host disease, allergic asthma

## Abstract

Plasmacytoid dendritic cells (pDCs) regulate innate and adaptive immunity. Neurotrophins and their receptors control the function of neuronal tissue. In addition, they have been demonstrated to be part of the immune response but little is known about the effector immune cells involved. We report, for the first time, the expression and immune-regulatory function of the low affinity neurotrophin receptor p75 neurotrophin receptor (p75NTR) by the antigen-presenting pDCs, mediated by toll-like receptor (TLR) 9 activation and differential phosphorylation of interferon regulatory factor 3 and 7. The modulation of p75NTR on pDCs significantly influences disease progression of asthma in an ovalbumin-induced mouse model mediated by the TLR9 signaling pathway. p75NTR activation of pDCs from patients with asthma increased allergen-specific T cell proliferation and cytokine secretion in nerve growth factor concentration-dependent manner. Further, p75NTR activation of pDCs delayed the onset of autoimmune diabetes in RIP-CD80GP mice and aggravated graft-versus-host disease in a xenotransplantation model. Thus, p75NTR signaling on pDCs constitutes a new and critical mechanism connecting neurotrophin signaling and immune response regulation with great therapeutic potential for a variety of immune disorders.

## Introduction

Plasmacytoid dendritic cells (pDCs) are a subset of dendritic cells that act at the interface of innate and adaptive immune responses. As antigen-presenting cells, pDCs modulate the differentiation and activity of T cell subsets, including T_H_1, T_H_2, T_H_17, and regulatory T cells. They are primarily linked to the host defense against pathogens and to pathogen adverse effects, particularly virus-induced interferon (IFN) α production (1, 2). pDCs are critically involved in the clearance of viral infections and are implicated in the pathophysiology of immune disorders (3, 4). In allergic diseases, IFNα secretion from pDCs is blocked by IgE disinhibiting T_H_2 and T_H_17 inflammatory reactions (5). Further, pDCs may prevent generation of effector T cells (4). A more complex field is evolving with regard to pDCs contribution in the pathology of type 1 diabetes (T1D): IFNα secretion by pDCs upon pancreotropic virus infection may be protective by reducing viral loads, whereas IFNα secretion may as well activate autoreactive T cells (6). In graft-versus-host disease (GvHD), pDCs presenting alloantigen may prime alloreactive T cells triggering GvHD (7). After allogeneic stem cell transplantation, diminished peripheral blood pDCs counts were associated with poorer odds of survival (8). Therefore, regulation of pDCs functions is an essential element in immune homeostasis.

The family of neuronal growth factors—the neurotrophins nerve growth factor (NGF), brain-derived neurotrophic factor (BDNF), neurotrophin 3, and neurotrophin 4/5—mediate the development, survival, and function of neuronal tissues. Neurotrophin signaling is mediated by tropomyosin-related kinase receptors (Trk) and the low affinity p75 neurotrophin receptor (p75NTR) (9). p75NTR belongs to the tumor necrosis factor receptor superfamily and consists of an extracellular, a transmembrane, and an intracellular domain. The extracellular domain contains four cysteine rich repeats that are crucial for NGF-p75NTR interaction (10). Transgenic disruption of exon III encoding cysteine rich repeat 2–4 destroyed the neurotrophin binding site in mice but produced viable animals (Ex3 mutant) without detectable expression of full length p75NTR (11). Interestingly, both wild-type and Ex3-mutant mice still express an alternative splice variant of p75NTR lacking exon III and thus a functional extracellular binding site for neurotrophins (12). Beside neuronal tissue, the expression of Trk receptors and p75NTR was detected in a variety of immune cells. B and T lymphocytes, macrophages, basophils, and mast cells express TrkA, and p75NTR expression was shown in B cells and mast cells [as reviewed in Ref. (13–15)]. The NGF-promoted effects comprise chemoattraction, activation and differentiation of neutrophils, T_H_2 lymphocyte proliferation, and B cell survival, among others (16). Furthermore, the neurotrophins and p75NTR have been demonstrated to play a pivotal role in the etiology of immune disorders. Bronchoalveolar lavage samples from patients with asthma contain high concentrations of neurotrophins, and mice with inactivated p75NTR fail to develop an ovalbumin (OVA)-induced asthma (17, 18). p75NTR-deficient mice with experimental autoimmune encephalitis exhibit enhanced inflammatory responses (19). Endogenous levels of NGF have been shown to be altered in human and experimental diabetes, while p75NTR was shown to regulate glucose homeostasis and insulin sensitivity in diabetes (20–22). Thus, it has been postulated that neurotrophins and p75NTR transmit signals directly to the immune cells.

In the present study, we report the discovery of a new role for p75NTR signaling during pDC-mediated immune responses applying models of an allergic asthma, an autoimmune diabetes type I (T1D) and a GvHD.

## Materials and Methods

### Animals

The p75NTR (Ex3)-mutant strain (B6.129S4-Ngfrtm1Jae/J) and the NSG mice (NOD.Cg-Prkdcscid Il2rgtm1Wjl/SzJ) were purchased from The Jackson Laboratory (11, 23). The OT-I (C57BL/6-Tg (TcraTcrb)1100Mjb/Crl) and the OT-II (C57BL/6-Tg (TcraTcrb)425Cbn/Crl) strain were purchased from Charles River (24, 25). Heterozygous RIP-CD80GP mice were generated by cross-breeding RIP-CD80 with RIP-lymphocytic choriomeningitis virus glycoprotein (LCMV-GP) mice as previously described (26). All mice were bred under pathogen-free conditions in the animal facility of the Technische Universitaet Dresden. Male and female mice 10–12 weeks of age were used for the experiments. All animal experiments were carried out in strict accordance with the German Animal Welfare Act. The protocol was approved by the Committee on Ethics of the Landesdirektion Dresden, Germany (Permit Number: 24-9168.11-1/2010-34, 24-9168.11-1/2013-32).

### Isolation of Human pDCs and T Cells

Plasmacytoid dendritic cells were isolated after donors granted informed consent with approval of the local ethics committee (EK 155052009). Cells were purified using magnetic cell separation (MACS, Miltenyi Biotec) according to the manufacturer’s instructions. For *in vitro* assays, pDCs and CD4^+^ T cells were purified using BDCA-4 MicroBeads and a CD4^+^ T cell Isolation Kit II, respectively. For transplantation, pDCs and T cells were purified using a pDC Isolation Kit II and a Pan T cell Isolation Kit, respectively. The purities of pDCs (BDCA-2^+^, BDCA-4^+^) and T cells (CD3^+^) were greater than 95% as assessed by flow cytometric analysis.

### Stimulation of Human pDCs *In Vitro*

Isolated pDCs (5 × 10^4^ cells/96-well cavity) were seeded in 200 µl of RPMI 1640 medium (Thermo Fisher Scientific) supplemented with 100 IU ml^−1^ penicillin, 100 µg ml^−1^ streptomycin, 2 mM l-glutamine, 10% (vol/vol) human serum and recombinant human (rh) IL-3 [10 ng ml^−1^; R&D Systems]. To determine the levels of secreted IFNα, pDCs were cultured for 12–14 h in the presence of ODN 2216 (CpG A; 0.26 µM; Thermo Fisher Scientific). To block p75NTR activity, the inhibitory peptide TAT-Pep5 (100 nM, Merck) was added. Recombinant human β-NGF (R&D Systems) was added at the indicated concentrations. The IFNα levels in supernatants were determined using an ELISA (eBioscience). To determine the levels of secreted IL-6, pDCs were stimulated with an antibody specific to human FcεRlα (250 ng ml^−1^; eBioscience) alone or in the presence of the TAT-Pep5 inhibitor. Recombinant human β-NGF was added at the indicated concentrations. After 14 h, the supernatant was collected to measure IL-6 levels using an ELISA (eBioscience).

### Autologous Coculture of Human pDCs and CD4^+^ T Cells *In Vitro*

CD4^+^ T cells and pDCs were isolated from an individual with an untreated, well-defined allergy. CD4^+^ T cells were stained with CFSE (1 µM; Thermo Fischer Scientific) for 8 min at 37°C followed by the addition of 1 ml warmed FCS. The cells were washed twice and counted directly before starting the coculture. Purified pDCs and CFSE-labeled T cells were cocultured (ratio 1:6) in a 96-well U-bottom plate at 37°C and in an atmosphere containing 5% (vol/vol) CO_2_. Each well contained 200 µl of RPMI 1640 medium supplemented with 100 IU ml^−1^ penicillin, 100 µg ml^−1^ streptomycin, 2 mM l-glutamine, 10% (vol/vol) human serum, and rh IL-3 (10 ng ml^−1^). Recombinant human β-NGF (5 or 25 ng ml^−1^) was added to the designated wells. Respective allergens were added to the coculture at 50 SBE U ml^−1^ (ALK-Abelló Arzneimittel). After 5 days of coculture, the plate was centrifuged at 270 × *g* for 5 min, and the supernatant was collected. The levels of T_H_1- and T_H_2-specific cytokines were measured using a cytometric bead array (CBA) assay (BD Biosciences), and the percentage of proliferating CD4^+^ T cells was determined by quantification of CFSE signal intensity using flow cytometry. CD4^+^ T cells with lower CFSE signals compared to initial CFSE staining signal were assumed as proliferating T cells and percentage of proliferating T cells were calculated in relation to all T cells (CD3^+^).

### Xenotransplantation GvHD Model

Recipient mice from the NSG strain received sublethal whole body irradiation using an Yxlon MaxiShot X-ray irradiator (Yxlon; 100 cGy), 20 h prior to human cell transplantation. Human pan T cells and human pDCs from five healthy donors were isolated as described above. Autologous pan T cells (2 × 10^6^/setup and donor) and pDCs (2 × 10^5^/setup and donor; ratio 10:1) were cultured together overnight in 200 µl RPMI 1640 complete medium containing rh IL-3 (10 ng ml^−1^) and human AB serum in the absence or presence of CpG A (0.26 µM) and rh β-NGF (25 ng ml^−1^). Next day, cell suspensions in 1× PBS (150 µl) were injected into the retro-orbital venous plexus using 25-gauge needles. For the first 3 weeks after transplantation, water was supplemented with neomycin (1.17 g l^−1^, Sigma-Aldrich). Mice were sacrificed when deterioration of health as recognized by weight loss (≥20% of starting weight), reduced activity, reduced pair grooming, inability to intake food, neurological malfunction, or self-mutilation appeared. Primary endpoint was overall survival with a maximal follow-up of 12 weeks after cell transplantation. Peripheral blood of the recipients was obtained every 1–2 weeks by venipuncture of the retro-orbital venous plexus using heparinized microcapillaries. Red blood cell lysis was performed using ACK lysis buffer (Thermo Fisher Scientific) according to manufacturer’s instructions. Distribution of human and murine cells in peripheral blood was analyzed by flow cytometry.

### Isolation of Murine Splenocytes

In order to isolate murine splenocytes, the spleen was dissected and a 70-µm cell strainer was used to generate single cell suspension from whole spleen. Cells were centrifuged for 8 min at 300 *g* at 4°C. Erythrocytes were removed using ACK lysis buffer according to manufacturer’s instructions. After the washing step, the cells were suspended in RPMI 1640 medium supplemented with 10% (vol/vol) FCS, 1 mM sodium pyruvate, 2 mM l-glutamine, 100 IU ml^−1^ penicillin, 100 µg ml^−1^ streptomycin, 10 mM HEPES buffer, and 0.1 mM β-mercaptoethanol.

### Generation of Mouse pDCs

Bone marrow (BM)-derived pDCs were generated *in vitro* as described previously (27). Briefly, BM cells were isolated from mice by flushing the femur and tibia. Erythrocytes were lysed using ACK lysis buffer according to manufacturer’s instructions. The remaining cells were washed and cultured at a density of 2 × 10^6^ cells ml^−1^ in RPMI 1640 medium supplemented with 10% (vol/vol) FCS, 1 mM sodium pyruvate, 2 mM l-glutamine, 100 IU ml^−1^ penicillin, 100 µg ml^−1^ streptomycin, 10 mM HEPES buffer, and 0.1 mM β-mercaptoethanol. To differentiate BM cells into pDCs, rh Flt3-L (100 ng ml^−1^; R&D Systems) was added to the cells. After 8 days, pDCs were enriched by removing the CD11b^+^ cells from the non-adherent cells, using CD11b MicroBeads (Miltenyi Biotec) according to manufacturer’s instructions. Dead cells were excluded using a dead cell removal Kit (Miltenyi Biotec). The purity of BM-derived pDCs (B220^+^, SiglecH^+^, mPDCA1^+^, CD11b^−^) was greater than 98% according to flow cytometric analysis.

### Stimulation of Mouse pDCs *In Vitro*

For all *in vitro* stimulation assays, 2 × 10^5^ cells were cultured at 200 µl per 96-well of RPMI 1640 complete medium in the presence of either ODN1585 (CpG A, 1 µM; Invivogen), ODN1826 (CpG B, 1 µM; Invivogen), lipopolysaccharide (LPS, 1 µg ml^−1^; Sigma-Aldrich), low endotoxin ovalbumin (OVA LE; 1 µg ml^−1^; Invivogen), or grade V ovalbumin (OVA V, 1 µg ml^−1^; Sigma-Aldrich). Control cells were left untreated. If not stated otherwise, recombinant murine (rm) β-NGF (100 ng ml^−1^; R&D Systems) was used according to previous studies of a mouse model of OVA-mediated allergic asthma (28). The production of mouse IFNα was measured 24 h after set-up using an ELISA (eBioscience). Murine IL-6 and TNFα levels were assessed using a CBA (BD Biosciences). The remaining cells were used for flow cytometric analysis.

### Coculture of Mouse pDCs and T Cells

Before coculture, purified BM-derived wild-type (WT) or Ex3 pDCs were incubated overnight with OVA V (100 µg ml^−1^) in the absence or presence of rm β-NGF (100 ng ml^−1^) and then washed and suspended in RPMI 1640 complete medium. Control cells were incubated without OVA V. Untreated CD4^+^ T and CD8^+^ T cells from the OT-II and OT-I strains, respectively, were isolated from mouse splenocytes using MACS Microbeads (CD4^+^ T cell Isolation Kit II, CD8α^+^ T cell Isolation Kit II, Miltenyi Biotec). CD4^+^ and CD8^+^ T cells were stained with CFSE (1 µM) according to manufacturer’s instructions. CFSE-labeled T cells and pDCs were cocultured (ratio 10:1) in 200 µl of RPMI 1640 complete medium in a 96-well culture plate at 37°C for 4 days. The culture plates were then centrifuged at 400 × *g* for 4 min at 4°C. Cytokine levels were measured using a CBA (BD Bioscience), and T cell proliferation was determined by flow cytometric analysis of CFSE signal intensity.

### Induction of Autoimmune Diabetes in Mice

Bone marrow-derived WT pDCs were stimulated for 2 h with CpG B (1 µM) and the lymphocytic choriomeningitis virus (LCMV) gp33-41 peptide (10 µM; Eurogentec) in the absence or presence of rm β-NGF (100 ng ml^−1^) in RPMI 1640 complete medium at 0.5 × 10^6^ cells ml^−1^. Cells were washed twice with 1× PBS. Finally, cell suspension (1.7 × 10^6^ pDCs in 150 µl 1× PBS) was injected intravenously into the tail vain of heterozygous RIP-CD80GP mice. Blood glucose levels were measured twice weekly using the Glucometer Elite (Bayer Diagnostics). Mice were sacrificed immediately after positive diagnosis of diabetes defined by two consecutive blood glucose levels above 250 mg dl^−1^. If not stated otherwise, mice were followed for 48 days post cell transplantation.

### Induction of OVA-Specific Allergic Asthma in Mice

Sensitization to OVA was performed as described by Lambrecht et al. with minor modifications (29). To induce allergic asthma, pDCs generated *in vitro* were first incubated with 100 µg ml^−1^ OVA V in the absence or presence of rm β-NGF (0.1 or 100 ng ml^−1^) for the next 24 h. Next, 1 × 10^6^ OVA V-loaded pDCs were injected intratracheally into the lungs of anesthetized mice using a 24-gauge i.v. cannula (BD Biosciences). Control animals received either the same amount of pDCs without OVA V or with PBS only. After 10 days, mice were exposed to an OVA V aerosol [1% (wt/vol)] for 30 min on three consecutive days to induce an allergic reaction. Animals were sacrificed 24 h after the last provocation, and the presence of an immune reaction was assessed according to the cell composition of the bronchoalveolar lavage fluid (BALF), proinflammatory cytokine levels, and lung histology.

### Analysis of BALF

Cells present in BALF were quantified using flow cytometry according to a modified method described previously (30). To minimize non-specific binding, cells were preincubated with FcR blocking reagent (Miltenyi Biotec). Among lymphocytes (FSC^low^/SSC^low^), the CD4^+^ T cells were designated as CD3^+^ CD4^+^, the CD8^+^ T cells as CD3^+^ CD8^+^, B cells as CD3^−^ B220^+^, and among granulocytes (FSC^low^/SSC^high^, Ly6G^+^) eosinophils were designated SiglecF^+^ CD11c^−^ and neutrophils as SiglecF^−^. Macrophages were defined as FSC^high^ highly autofluorescent CD11c^+^ F4/80^+^. Cytospins were stained using Pappenheim’s method. Staining patterns are as follows: cell nuclei are stained purple to violet, plasma lymphocytes are light blue, monocytes/macrophages are violet, the granules of eosinophils are red-brown, and neutrophils are light violet. ELISA (eBioscience) was used to quantify the levels of IL-4, IL-5, and IL-13 according to manufacturer’s instructions.

### Histological Analyses

Lungs were perfused with 1× PBS and fixed in 4% (vol/vol) formaldehyde. 4-µm sections of paraffin-embedded lungs were stained with PAS to quantify inflammation and Goblet cell hyperplasia. The number of Goblet cells in stained lung sections was determined using a 5-point scoring system as follows: grade 0, none detected in the epithelium; grade 1 < 25%; grade 2, 25–50%; grade 3, 51–75%; and grade 4 > 75%.

### Flow Cytometry

Flow cytometric analyses were conducted using an LSR II flow cytometer (BD Biosciences). Data were evaluated using FlowJo software (Version 7.6.5; Flowjo). All antibodies against human or mouse cells were used at appropriate dilutions, as determined by previous titration. Doublet discrimination was carried out and non-viable cells were excluded by 4,6 diamidino-2-phenylindole (DAPI) staining (Sigma-Aldrich). Mouse cells were characterized using the antibodies as follows: CD3-V500, CD4-V450, CD11c-APC Cy7, SiglecF-AF647, Ly6G-FITC, CD11b-V500, B7-1-V450, B7-2-PE Cy7, CD62L-FITC (BD Biosciences); mPDCA1-APC, B220-PE (Miltenyi Biotec); CD8-PE Cy7, F4/80-PerCP, SiglecH-PE, TLR4-AF488, TLR9-FITC, MHC-I-FITC, MHC-II-V450, PD-L1-PerCP, ICOS-L-PE, OX40L-APC (eBioscience); TLR7-PE (Abcam); p75NTR-AF488 (Advanced Targeting Systems), CD45-Pacific Blue (Biolegend), and panTrk-FITC (Cell Signaling Technology). Human cells were characterized using the antibodies as follows: TrkA-PE (R&D Systems); BDCA-2-FITC, BDCA-4-PE, p75NTR-APC, p75NTR-FITC, p75NTR-PE (Miltenyi Biotec); CD45-V500, CD3-PE, CD4-FITC, CD8-PerCP, FcεRIα-FITC, IL-3R-PE Cy7, CD184-PE Cy7, MHC-I-PE Cy7, MHC-II-PE, CD80-V450, CD86-PE, CD83-V500, OX40L-V500, PD-L1-PE Cy7, CCR7-V450, CCR9-APC (BD Biosciences), CD3-APC eFluor780, CD4-APC, CD8-PE Cy7, CD25-PE (eBioscience), and CD69-PerCP Cy5.5 (BioLegend).

### Western Blotting

Whole-cell lysates were prepared from BM-differentiated pDCs. For signaling pathway analyses, the cells were first cultured for 5 h in the absence or presence of rm β-NGF (100 ng ml^−1^) and treated for 15 min with CpG A or CpG B (1 µM) each. Then, the cells were immediately collected for protein isolation using complete RIPA buffer containing MiniComplete Protease inhibitor cocktail and PhosSTOP Phosphatase inhibitor cocktail (Roche Diagnostics). The reduced and denatured protein samples (10 µg) were loaded onto a NuPAGE 4–12% Bis-Tris gel (Thermo Fisher Scientific), electrophoresed, and transferred electrophoretically to a nitrocellulose membrane. Immunodetection was conducted using the primary antibody specific to following mouse proteins generated in rabbit: p75NTR (D4B3), TrkA, MyD88, Phospho-IRF3, Phospho-IRF7, Phospho-IKKα/β, Phospho-c-Jun (Ser63) II (Cell Signaling Technology); TRAF6 (Thermo Fisher Scientific); GAPDH (GeneTex). The antibodies were diluted 1:1,000, except for the GAPDH antibody (which was diluted 1:10,000). Incubation with primary antibody was followed by incubation with HRP-conjugated secondary anti-rabbit antibody (GE Healthcare) before development using Lumi-Light Plus western blot detection system (Roche Diagnostics).

### Statistical Analyses

Unless stated otherwise, data generated from *in vivo* experiments were analyzed with one- or two-way ANOVA models. In case of comparing mean values of only two groups, Welch’s *t*-test was used. Where necessary, a logarithmic (log) transformation (natural logarithm, ln, base *e*) was used to stabilize the variance in the different groups. Occurrence of autoimmune diabetes was analyzed with a standard log-rank test comparing two groups. Survival time and human T cell distribution in the GvHD xenotransplantation context was described with a Cox proportional hazards regression model. For OVA-induced asthma model, the ordinal Goblet cell scores on lung sections were analyzed *via* a proportional odds model (31). The OVA-uptake measurements over time were analyzed with a repeated-measure ANOVA. Log-scale data from experiments using human materials to assess the influence of NGF and p75NTR expression level on the secretion of IFNα and IL-6, and the proliferation of T cells were evaluated using mixed effect models to accommodate for the human donor effect (32). For human cytokine data an ordinary two-way ANOVA was performed, as the number of donors was too small to be modeled in the context of mixed effect models. *Post hoc* pairwise comparisons in the analyses were done either with Tukey’s honest significance test based on a multivariate *t*-distribution for all-pair comparisons or with Dunnett’s test for multiple comparisons against a control. The number of experimental and technical replicates is shown in the figure legends. *p*-Values of less than 0.05 were considered statistically significant. The statistical analyses were done with GraphPad Prism (Version 5.04; GraphPad Software) and R (Version 3.1.2) (33).

## Results

### p75NTR Modulates the Innate Immune Response of Murine pDCs

**Figure 1:**
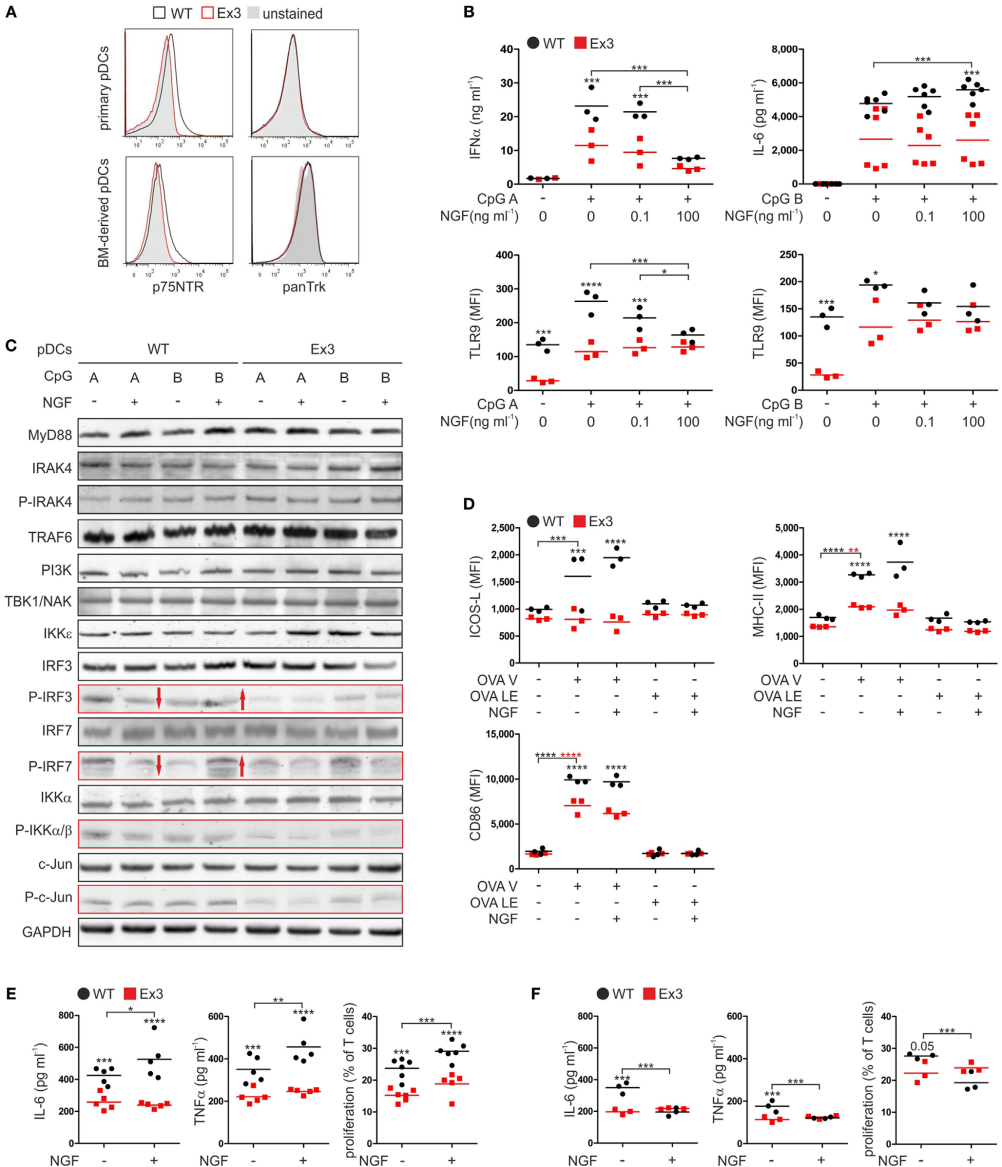
Nerve growth factor (NGF) modulates the innate immune response of murine plasmacytoid dendritic cells (pDCs) *in vitro*. **(A)** Expression of p75 neurotrophin receptor and tropomyosin-related kinase receptors on primary pDCs isolated from spleen and bone marrow (BM)-derived pDCs derived from wild-type (WT; black line) mice or Ex3 mutant mice (red line). Unstained controls are displayed in gray. **(B)** Secretion of IFNα and IL-6 and TLR9 protein levels by BM-derived WT or Ex3 pDCs stimulated overnight with CpG A or CpG B in the absence or presence of NGF. **(C)** Total protein and protein phosphorylation levels (P) of toll-like receptor signaling pathway members in BM-derived WT or Ex3 pDCs stimulated overnight with CpG A or CpG B in the absence or presence of NGF. Evident differences between WT and Ex3 pDCs are indicated by the red-bordered rectangles. Evident changes in phosphorylation upon addition of NGF to WT pDCs are indicated by red arrows. **(D)** Expression of ICOS-L, MHC-II, and CD86 of BM-derived WT or Ex3 pDCs stimulated overnight with endotoxin-free OVA (OVA LE) or grade-V OVA (OVA V) in the absence or presence of 100 ng ml^−1^ NGF. **(E)** IL-6 and TNFα levels, and T cell proliferation of cocultures of primary CD4^+^ OT-II-derived T cells and BM-derived, OVA V-pulsed WT, or Ex3 pDCs in the absence or presence of 100 ng ml^−1^ NGF. **(F)** IL-6 and TNFα levels, and T cell proliferation of cocultures of primary CD8^+^ OT-I-derived T cells and BM-derived, OVA V-pulsed WT, or Ex3 pDCs in the absence or presence of 100 ng ml^−1^ NGF. In panels **(A,C)**, representative images are shown. BM-derived WT-pDCs and Ex3 pDCs, respectively, are indicated in panels **(B,D–F)** as black dots and red squares. In panels **(B,D–F)**, number of independent experiments are displayed as single marks in scatter plots with mean values indicated by the short horizontal lines. MFI, mean fluorescence intensity (*p < 0.05, ***p < 0.005, ****p < 0.0001).

Harvesting splenocytes from WT mice for flow cytometric analyses, we detected a cell population that expressed p75NTR without expressing high affinity neurotrophin Trk receptors (Figure 1A). Analyzing the surface marker expression profile of this p75NTR positive cell population in more detail we identified them as pDCs (Figure S1A in Supplementary Material).

To assess the role of p75NTR in function of murine pDCs, we applied the Ex3 mutant mouse strain. In this strain, a cassette is introduced in the third exon of the gene encoding p75NTR suppressing the expression of functional p75NTR (11). Except for their difference in p75NTR cell-surface expression, primary pDCs from spleen of WT and Ex3 mice exhibited no differences in the levels of their cell-surface markers and showed no expression of any of the high affinity neurotrophin receptors (Figure 1A; Figure S1A in Supplementary Material). Further, BM-derived murine pDCs did not exhibit any difference in cell-surface marker expression compared with that of primary pDCs (Figure 1A; Figure S1A in Supplementary Material).

Regarding the pDC function in innate immune response, we determined the expression of toll-like receptors (TLR) 4, 7, and 9 of receptor-specific stimulated pDCs (Figure S1B in Supplementary Material). TLR4, which is typically expressed on conventional dendritic cells, was not expressed by WT or Ex3 pDCs. Only a small subset of WT and Ex3 pDCs expressed TLR7 increased further by exposing cells to type A (CpG A) or type B (CpG B) CpG oligodeoxynucleotides. Similarly, administration of CpG A or CpG B markedly increased the percentage of both WT and Ex3 pDCs that expressed TLR9. Furthermore, OVA with grade-V (OVA V) purity and LPS substantially increased the levels of TLR7 and TLR9.

We next tested the influence of NGF on the secretion of cytokines by WT and Ex3 pDCs stimulated with CpG A or CpG B. When compared with Ex3 pDCs, CpG A-stimulated WT pDCs secreted significantly higher levels of IFNα (*p* = 0.003; Figure 1B). Concomitant addition of NGF attenuated IFNα secretion by WT pDCs in a significant, concentration-dependent manner (100 ng ml^−1^ NGF: *p* = 0.0003). NGF had no significant effect on mean IFNα levels of Ex3 pDCs. NGF alone did not induce a secretion of IFNα by WT or Ex3 pDCs (Figure S1C in Supplementary Material). An increased amount of the proinflammatory cytokine IL-6 was produced by pDCs treated with CpG B. In the presence of NGF (100 ng ml^−1^), WT pDCs secreted significantly higher levels of IL-6 whereas Ex3 pDCs did not (*p* < 0.0001; Figure 1B). WT pDCs treated with CpG A expressed significantly reduced levels of TLR9 in response to NGF (100 ng ml^−1^ NGF: *p* = 0.0011), in a dose-dependent manner (Figure 1B). Interestingly, Ex3 pDCs expressed significantly lower basal levels of TLR9 (*p* = 0.0046) and significantly lower levels of TLR9 when treated with CpG A (*p* < 0.0001) or CpG B (*p* = 0.018; Figure 1B).

To investigate the mechanisms underlying the observed effects, we analyzed pDCs using western blotting analysis (Figure 1C). WT and Ex3 pDCs expressed comparable levels of MyD88, IRAK4, TRAF6, PI3K, TBK1/NAK, and IKKε kinase, which are involved in signaling pathways activated by CpG A and CpG B. Interestingly, treatment with CpG A increased the phosphorylation of IRF3, IRF7, IKKα/β, and c-Jun in WT pDCs as compared to Ex3 pDCs (Figure 1C). The total protein concentrations of IRF3, IRF7, IKKα/β, and c-Jun were comparable among the samples. NGF attenuated CpG A-induced phosphorylation of the transcription factors IRF3 and IRF7 in WT pDCs, while CpG B-induced phosphorylation of IRF3 and IRF7 was augmented by NGF treatment (Figure 1C). NGF did not alter the levels of CpG-induced phosphorylation of IRF3 and IRF7 expressed by Ex3 pDCs.

In conclusion, p75NTR alters the TLR9 specific IFNα secretion of pDC *via* an altered activation of the interferon regulatory factor (IRF) signaling pathway in NGF-treated pDCs.

### p75NTR Modulates the Ability of Murine pDCs to Stimulate T Cells

Antigen presentation and stimulation of T cells are central functions of pDCs in adaptive immune response. To study the effect of p75NTR on pDC driven antigen presentation, we incubated WT and Ex3 pDCs with different OVA formulations (Figure 1D). Treating WT and Ex3 pDCs with OVA V significantly increased the surface expression of MHC-II (WT: *p* = 0.0001, Ex3: *p* = 0.0013) and CD86 (WT: *p* < 0.0001, Ex3: *p* < 0.0001), although expression was significantly higher for both molecules in WT pDCs. Only WT pDCs expressed significantly higher levels of cell-surface ICOS-L in response to OVA V treatment (*p* = 0.0004). The addition of NGF in combination with OVA V did not alter the expression of these molecules on the surface of WT or Ex3 pDCs. Low endotoxin OVA (OVA LE) had no significant effect on the cell-surface expression of molecules required for costimulation and antigen presentation.

We next determined the ability of WT and Ex3 pDCs to stimulate T cells. For this purpose, we purified T cells from transgenic OT-I and OT-II mice that express OVA-specific CD8 or CD4 pairing T cell receptors, respectively. Compared to cocultures with OVA V-loaded Ex3 pDCs, cocultures with OVA V-loaded WT pDCs contained significantly higher levels of IL-6 (CD4^+^ OT-II T cells: *p* = 0.0039, CD8^+^ OT-I T cells: *p* = 0.0001) and TNFα (CD4^+^ OT-II T cells: *p* = 0.0031, CD8^+^ OT-I T cells: *p* = 0.0023). The proliferation of CD4^+^ OT-II (*p* = 0.0001) and CD8^+^ OT-I (*p* = 0.051) T cells were increased (Figures 1E,F). In cocultures of CD4^+^ OT-II T cells and OVA V-loaded WT pDCs, IL-6 (*p* = 0.042) and TNFα secretion (*p* = 0.0071), and T cell proliferation (*p* = 0.0046) were significantly enhanced in the presence of NGF. In contrast, IL-6 (*p* = 0.0001) and TNFα (*p* = 0.0026) production, as well as T cell proliferation (*p* = 0.0029) were attenuated by NGF treatment in cocultures of CD8^+^ OT-I T cells and OVA V-loaded WT pDCs. A comparable effect of NGF on CD4^+^ OT-II or CD8^+^ OT-I cocultures with OVA V-loaded Ex3 pDCs was not observed.

In conclusion, pDC expressed co-stimulatory and antigen-presentation surface proteins in a p75NTR dependent manner. Upon addition of NGF, pDC stimulated the proliferation and activity of antigen-specific CD4^+^ T cells, whereas CD8^+^ T cells displayed an attenuated behavior.

### pDCs Expressing p75NTR Induce OVA-Mediated Allergic Asthma in Mice

To address the role of p75NTR expression by pDCs in immune disorders *in vivo*, we applied a T_H_2-prone murine model of OVA-induced allergic asthma. We sensitized WT and Ex3 mice through the intratracheal application of OVA V-pulsed WT or Ex3 pDCs and induced an allergic reaction by subsequent exposure to OVA aerosol.

**Figure 2:**
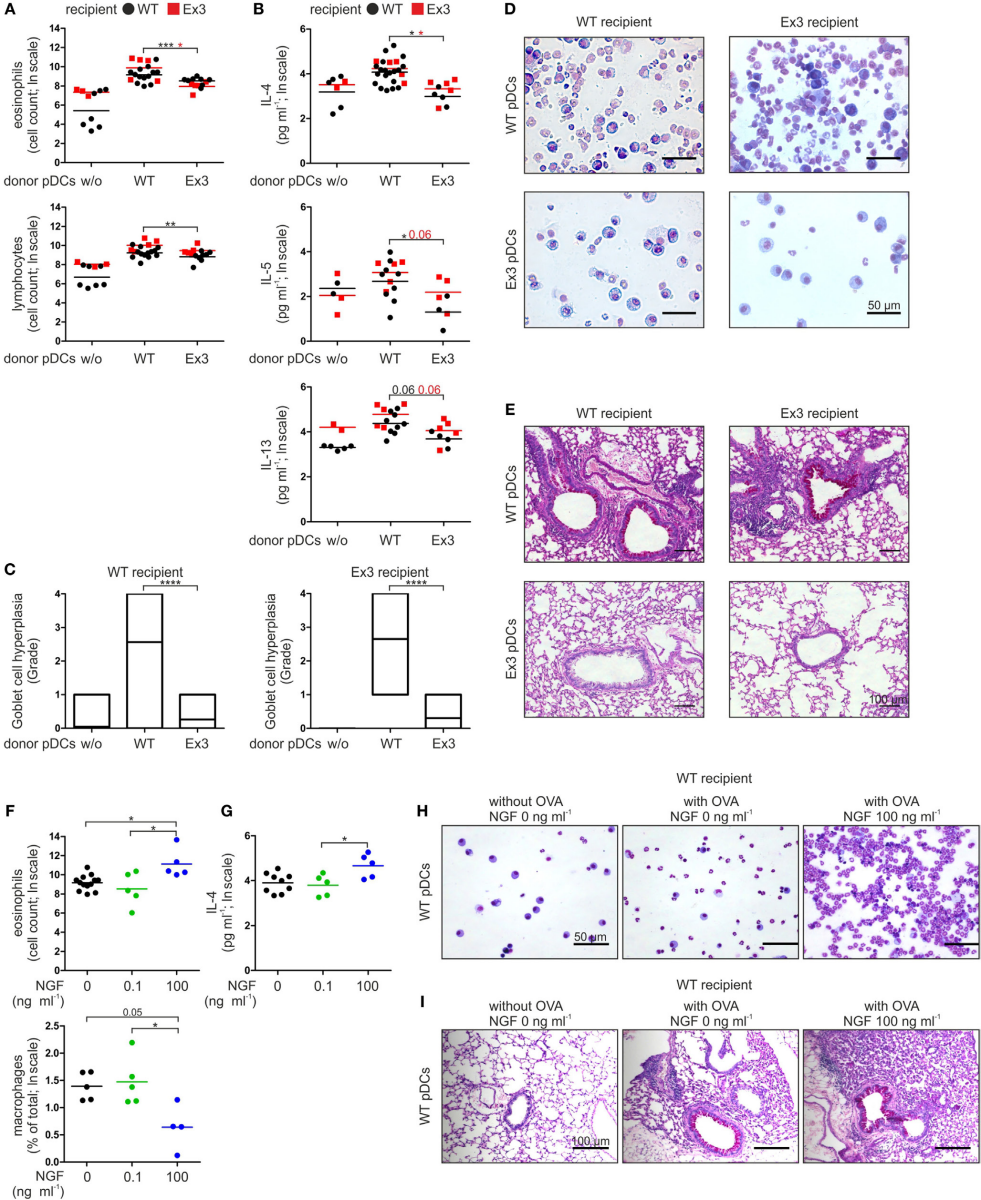
Stimulation of p75 neurotrophin receptor-expressing plasmacytoid dendritic cells (pDCs) with nerve growth factor (NGF) aggravates allergic responses in mice. **(A)** Cellular composition of bronchoalveolar lavage fluid (BALF) harvested from wild-type (WT) or Ex3 mice with ovalbumin (OVA)-induced allergic asthma instilled with WT pDCs, Ex3 pDCs, or no cells. **(B)** Levels of IL-4, IL-5, and IL-13 in BALF described in panel **(A)**. **(C)** Quantification of Goblet cell hyperplasia using a 5-point scoring system as described in Section “Materials and Methods.” Twenty-three bronchi from five recipient mice of each treatment were analyzed. **(D)** Pappenheim’s-stained cytospins from BALF. Cell nuclei are purple to violet, plasma lymphocytes are light blue, monocytes/macrophages are purple, and the granules of eosinophils and neutrophils are red-brown and light purple, respectively. **(E)** PAS-stained lung sections show purple staining of mucus-producing Goblet cells. **(F)** Cellular composition of BALF harvested from WT mice with OVA-induced allergic asthma instilled with OVA-loaded WT pDCs pretreated without or with NGF. **(G)** IL-4 level in BALF described in panel **(F)**. **(H)** Cytospins stained according to Pappenheim’s method of BALF described in panel **(F)**. **(I)** PAS-stained lung sections of mice described in panel **(F)** show purple staining of mucus-producing Goblet cells. In panels **(D,E,H,I)** representative images are shown. Bone marrow-derived WT-pDCs and Ex3 pDCs, respectively, are indicated in panels **(A,B)** as black dots and red squares. In panels **(F,G)**, different treatments of WT pDCs are indicated by black dots (no NGF), green dots (0.1 ng ml^−1^ NGF), and blue dots (100 ng ml^−1^ NGF). Data in panels **(A,B,F,G)** are shown as scatterplots on log scale, and the mean log-values of one to three independent experiments (one to five animals per group) are indicated by the short horizontal lines (*p < 0.05, **p < 0.01, ***p < 0.005, ****p < 0.0001).

Wild-type and Ex3 mice treated with OVA V-loaded WT pDCs exhibited typical symptoms of allergic asthma upon exposure to the OVA V aerosol. The number of lymphocytes and eosinophils (Figures 2A,D) and the levels of the T_H_2 cytokines IL-4, IL-5, and IL-13 (Figure 2B) were increased in BALF. WT and Ex3 mice sensitized with OVA V-loaded Ex3 pDCs exhibited significantly lower amounts of eosinophils (WT mice: *p* = 0.00016, Ex3 mice: *p* = 0.018) and lymphocytes (WT mice: *p* = 0.0098), and secreted reduced amounts of the proinflammatory cytokines IL-4 (WT mice: *p* = 0.018, Ex3 mice: *p* = 0.018) and IL-5 (WT mice: *p* = 0.049) in the BALF compared with mice treated with OVA-loaded WT pDCs (Figures 2A,B,D). Perivascular inflammation and Goblet cell hyperplasia in the lung were reduced in mice treated with Ex3 pDCs, compared with mice treated with WT pDCs (WT mice: *p* < 0.0001, Ex3 mice: *p* < 0.0001; Figures 2C,E). Interestingly, WT and Ex3 mice differed significantly in the number of lymphocytes (*p* = 0.049) and secreted IL-5 (*p* = 0.020) in the BALF of animals instilled with WT pDCs.

In conclusion, the inability of Ex3 mice to develop an allergic asthma can be allocated to the missing p75NTR expression on pDC. Further, formation of antigen-specific inflammation of lung tissue solely depends on transplantation of antigen-presenting pDC with native p75NTR expression.

### NGF Augments pDC-Mediated Allergic Asthma in a p75NTR-Dependent Manner

Based on our observations of a pDC-mediated and p75NTR-dependent immune response in mice, we determined the effect of NGF on WT pDCs in experimentally induced asthma. For this purpose, we incubated WT pDCs with OVA V in the absence or presence of NGF (0.1 or 100 ng ml^−1^) before intratracheal instillation into WT mice. When OVA V-uptake by WT pDCs occurred in the presence of 100 ng ml^−1^ NGF, the number of eosinophils (compared to 0 ng ml^−1^ NGF: *p* = 0.026, compared to 0.1 ng ml^−1^ NGF: *p* = 0.027) was significantly increased in the BALF, whereas the percentage of macrophages (compared to 0.1 ng ml^−1^ NGF: *p* = 0.037) was significantly reduced (Figures 2F,H). Moreover, instillation of OVA V-loaded WT pDCs treated with 100 ng ml^−1^ NGF led to significantly increased levels of IL-4 (compared to 0.1 ng ml^−1^ NGF: *p* = 0.043) in the BALF compared to instillation with pDCs pulsed with OVA V in the absence or presence of 0.1 ng ml^−1^ NGF (Figure 2G), whereas no significant differences were observed for IL-5 and IL-13. Lung sections from mice that received OVA V-loaded and NGF-stimulated WT pDCs exhibited increased perivascular inflammation and enhanced mucus production (Figure 2I).

In conclusion, ligand-specific activation of p75NTR on pDC controls the severity of an antigen-specific allergic asthma in mice in a concentration-dependent manner.

### NGF Alleviates pDC-Mediated Autoimmune Diabetes in Mice

Based on our observations in the T_H_2-prone murine model of an allergic asthma, we studied influence of pDC-specific activation of p75NTR on a T_H_1-prone mouse model of autoimmune diabetes. In this RIP-CD80GP model, islet β-cells express the neo-self-antigen LCMV-GP and the co-stimulatory molecule CD80 under control of rat insulin promotor construct.

**Figure 3:**
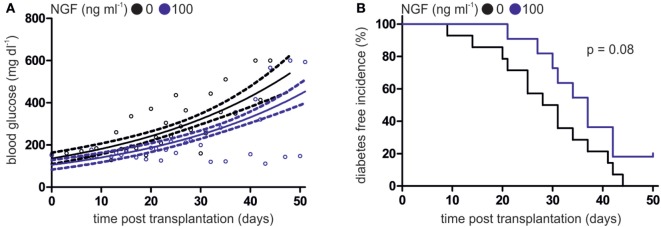
Nerve growth factor (NGF) alleviates plasmacytoid dendritic cell (pDC)-mediated autoimmune diabetes *in vivo*. **(A)** Glucose levels in peripheral blood of heterozygous RIP-CD80GP mice transplanted with murine pDCs. Murine pDCs were stimulated with CpG A and lymphocytic choriomeningitis virus peptide gp33–41 in the absence (black dots) or presence (blue dots) of NGF. Dots indicate single measurements. Correlation (continuous line) and confidence interval (95%; dashed lines) of both treatments are depicted. **(B)** Diabetes-free incidence of **(A)** is shown. Diabetes defined by two consecutive blood glucose levels above 250 mg dl^−1^. In panels **(A,B)** data of three independent experiments with two to five animals per group are shown.

After incubation with CpG B and LCMV gp33–41 peptide in the absence or presence of NGF, WT pDCs were transplanted into heterozygous RIP-CD80GP mice and the development of diabetes was determined by consecutive blood glucose measurement. Mice transplanted with NGF-treated WT pDCs displayed a delayed increase of blood glucose levels (Figure 3A) and later diabetes onset (Figure 3B) compared to mice transplanted with WT pDCs without preceding NGF treatment. The median diabetes-free incidence was prolonged by 7.5 days upon pre-treatment of transplanted WT pDCs with NGF (with NGF: 37 days; without NGF: 29.5 days; *p* = 0.08; Figure 3B). Two out of 11 (~18%) mice transplanted with NGF-treated pDCs developed no diabetes within 51 days of observation, while all mice transplanted with WT pDCs without preceding NGF treatment developed diabetes within 44 days (Figure 3B).

In conclusion, development and progression of antigen-specific autoimmune diabetes in mice is influenced by degree of p75NTR activation on pDC.

### p75NTR Signaling Modulates the Activity of Human pDCs

**Figure 4:**
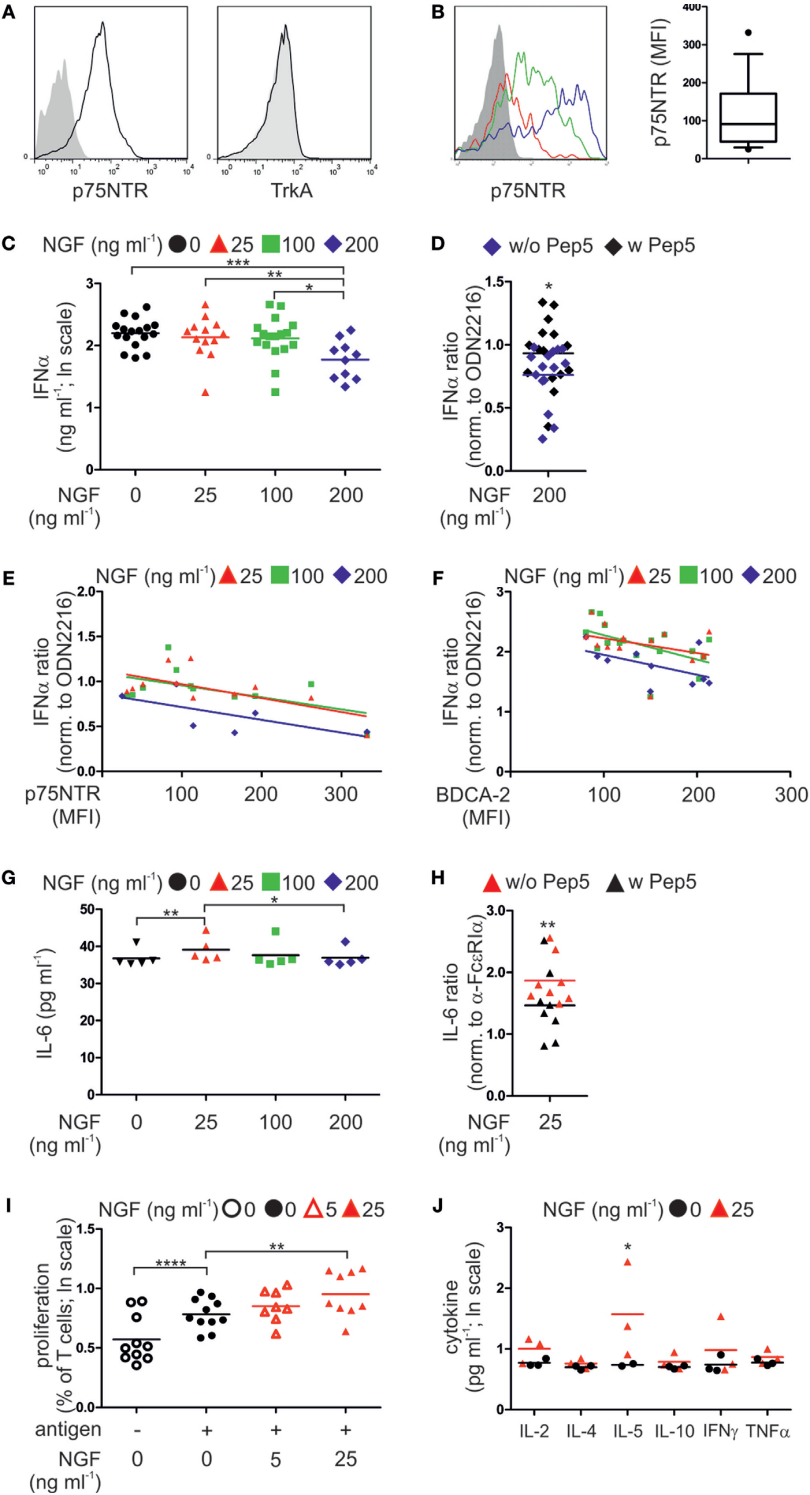
p75 neurotrophin receptor (p75NTR) is expressed by and modulates the activity of human plasmacytoid dendritic cells (pDCs). **(A)** Expression of p75NTR and TrkA receptor by human pDCs (black line) compared with unstained control (filled gray). **(B)** p75NTR expression by primary human pDCs from three different donors (red, green, and blue lines; gray, unstained control). The graph summarizes p75NTR levels (17 donors). **(C)** IFNα secretion by pDCs (17 donors) stimulated with CpG ODN2216 in the presence of nerve growth factor (NGF). **(D)** IFNα secretion by ODN2216-stimulated pDCs (15 donors) in the presence of NGF and the p75NTR inhibitory peptide Pep5. **(E)** IFNα secretion by pDCs (12 donors), normalized to treatment with ODN2216 alone, and correlated with p75NTR cell-surface expression. **(F)** IFNα secretion by pDCs (17 donors), normalized to treatment with ODN2216 alone, and correlated with BDCA-2 cell-surface expression. **(G)** IL-6 secretion by pDCs (five donors) stimulated overnight with an FcεRIα-specific IgE-crosslinking antibody and incubated with increasing NGF concentrations. **(H)** IL-6 secretion by pDCs (eight donors), normalized to those of antibody treatment only, stimulated overnight with an FcεRIα-specific, IgE-crosslinking antibody in the presence of NGF (25 ng ml^−1^) and the p75NTR inhibitory peptide Pep5. **(I)** Cell proliferation of CD4^+^ T cells from four allergic donors cocultured with autologous pDCs in the absence or presence of the patient specific antigen and NGF. **(J)** Level of proinflammatory cytokines in coculture of three allergic donors as described in panel **(I)**. Data in panels **(D,E,F–H)** are shown as scatterplots, and the mean values are indicated by the short horizontal lines. Data in panels **(C,I,J)** are shown as scatterplots on log scale, and the mean log-values are indicated by the short horizontal lines. In panels **(A,B)**, representative images are shown. MFI, mean fluorescent intensity; *p < 0.05, **p < 0.01, ***p < 0.005, ****p < 0.0001.

To determine whether our findings in mice apply to humans, we analyzed the expression of p75NTR by human pDCs. Human pDCs express the p75NTR but not the high affinity NGF receptor TrkA (Figure 4A; Figure S2A in Supplementary Material). However, levels of p75NTR vary greatly among pDCs harvested from individual human donors (Figure 4B).

According to our experiments using murine pDCs, isolated primary human pDCs from healthy donors were stimulated *ex vivo* by the TLR9 ligand type A CpG ODN2216 or the negative control ODN2243. When human pDCs were incubated with the negative control CpG ODN2243 NGF did not show any influence on IFNα secretion (Figure S2B in Supplementary Material). In contrast, increasing doses of NGF diminished the levels of IFNα secretion of human pDCs stimulated with ODN2216 in a negative, linearly dependent manner (*p* = 0.00078). The greatest significant attenuation occurred in the presence of the highest NGF concentration (200 ng ml^−1^, *p* = 0.0006; Figure 4C). Addition of the p75NTR-specific, inhibitory peptide TAT-Pep5 to NGF-treated pDCs significantly restored IFNα secretion, indicating that the NGF-induced inhibition of the IFN response was mediated through p75NTR (*p* = 0.047; Figure 4D). Further, the levels of p75NTR expressed by pDCs correlated with the effects of NGF on IFNα secretion in a significant negative and linear-dependent manner (*p* = 0.0028; Figure 4E). Higher levels of p75NTR were accompanied by a further decrease in IFNα secretion in response to the addition of NGF. Interestingly, also the levels of BDCA-2 expressed by pDCs correlated with the effects of NGF on IFNα secretion in a significant negative and linear-dependent manner (*p* = 0.0013; Figure 4F). We next studied the effect of NGF on pDCs treated with an FcεRIα-specific IgE-crosslinking antibody. The lowest NGF concentration (25 ng ml^−1^) was the most effective for increasing IL-6 secretion (*p* = 0.0078; Figure 4G). Inhibition of p75NTR using TAT-Pep5 significantly attenuated the effect of NGF on IL-6 secretion, indicating that this effect was specific for p75NTR (*p* = 0.0059; Figure 4H).

To analyze the influence of p75NTR signaling on the capacity of pDCs to stimulate T cells, we isolated and cocultured pDCs and T cells from allergic patients. In the presence of patient-specific allergens, the proliferation of T cells increased significantly (*p* < 0.0001; Figure 4I). Allergen-induced T cell proliferation was significantly further increased by NGF (25 ng ml^−1^, *p* = 0.0086) in a significant linear-dependent manner (*p* = 0.0018; Figure 4I). Quantification of cytokines revealed that NGF (25 ng ml^−1^) significantly increased the secretion of IL-5 (*p* = 0.039; Figure 4J).

In conclusion, also human pDC express p75NTR and display a ligand-specific alteration in innate and adaptive immune function of pDC.

### NGF Deteriorates GvHD

Human pDCs have been described as a predictive marker for the outcome of allogeneic stem cell transplantation and the development of GvHD (8, 34). To investigate the role of p75NTR signaling in human pDCs *in vivo*, we transplanted autologous, cocultured pan T cells and pDCs of five different, healthy human donors into immunodeficient NSG mice. During coculture cells were pre-treated without or with CpG A (0.26 µM) or NGF (25 ng ml^−1^), or both.

**Figure 5:**
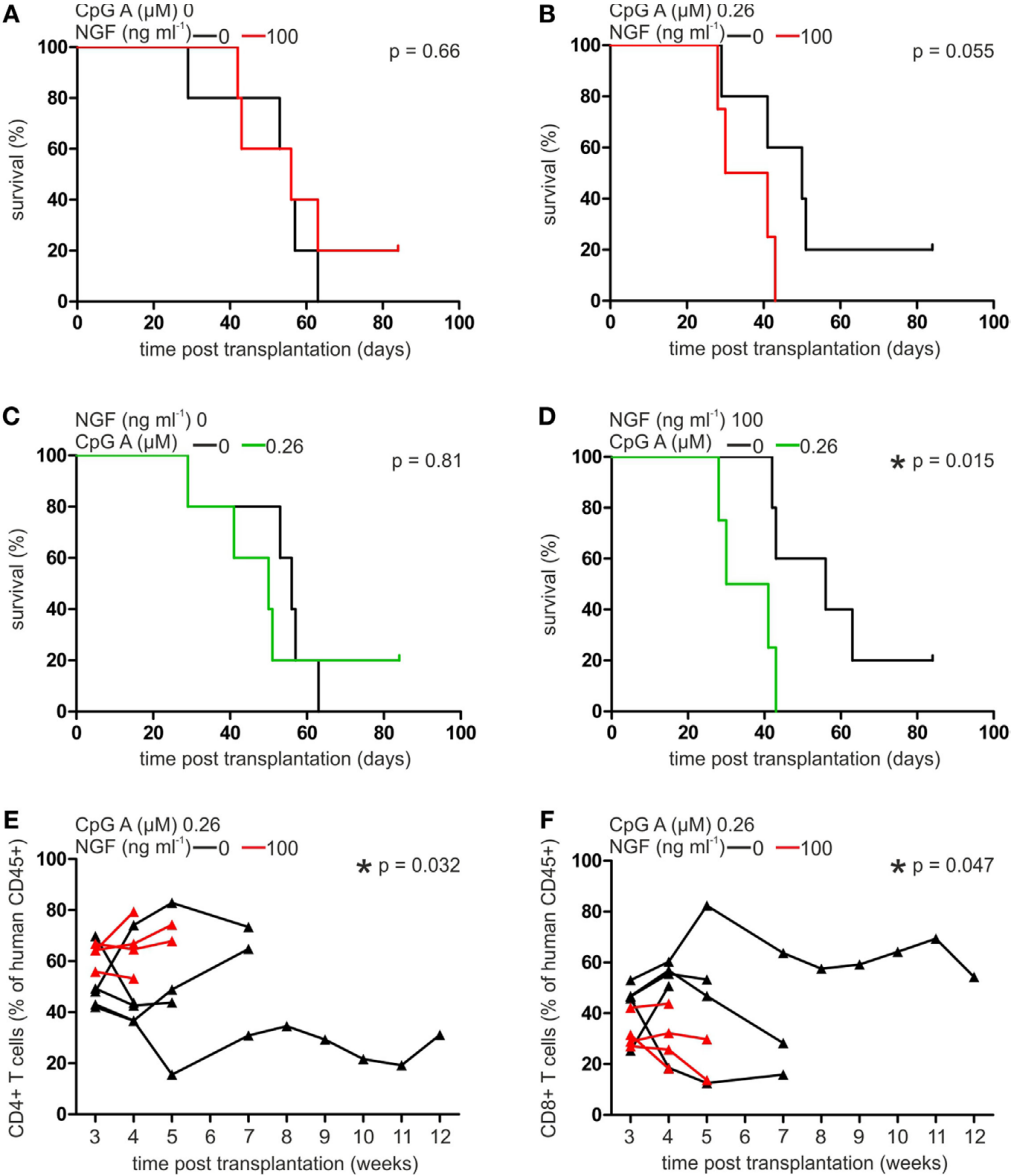
Nerve growth factor (NGF) aggravates plasmacytoid dendritic cell (pDC)-mediated graft-versus-host reaction in xenotransplantation model. Survival curve of NSG mice transplanted with autologous pan T cells and pDCs stimulated without or with NGF in the absence **(A)** or presence **(B)** of CpG A. Survival curve of NSG mice transplanted with autologous pan T cells and pDCs stimulated without **(C)** or with **(D)** NGF in the absence or presence of CpG A. Proportion of human CD45^+^ CD4^+^ **(E)** and CD8^+^ T cells **(F)** in peripheral blood of NSG mice transplanted with autologous pan T cells and pDCs stimulated without or with NGF in the presence of CpG A. In panels **(A,B,E,F)** data of five experiments each one independent, healthy human donor are indicated by individual horizontal black (without NGF treatment) or red (with NGF treatment) lines. Data of five experiments each one independent, healthy human donor are indicated in panels **(A,B)** by horizontal black (without NGF treatment) or red (with NGF treatment) lines, and in panels **(C,D)** by horizontal black (without CpG A treatment) or green (with CpG A treatment) lines, and in panels **(E,F)** are indicated by individual horizontal black (without NGF treatment) or red (with NGF treatment) lines and measuring points are indicated by black (without NGF treatment) or red (with NGF treatment) triangles (*p < 0.05).

Comparing the mean survival rate of NSG mice receiving pDCs and T cells without previous CpG A treatment, no significant difference was detectable when cells were cultured in absence or presence of NGF (without NGF: 51.6 ± 13.1, with NGF: 57.6 ± 17.2 days post transplantation; Figure 5A). Mice transplanted with CpG A-treated pDCs and T cells showed a clearly reduced mean survival rate close to statistical significance when NGF was present during coculture (without NGF: 51.0 ± 20.5, with NGF: 35.5 ± 7.8 days post transplantation; *p* = 0.055; Figure 5B). This observed relation between NGF and CpG A is confirmed by a significantly reduced mean survival of NSG mice transplanted with NGF and CpG A-treated cells (35.5 ± 7.8 days post transplantation) compared to mice transplanted with cells treated only with NGF (57.6 ± 17.2 days post transplantation; *p* = 0.015). Mice transplanted with cells that received no NGF showed no different survival rate independent from CpG A treatment (Figures 5C,D). NSG mice transplanted with T cells only displayed no difference in mean survival rate whether they were pre-treated without or with NGF.

Analyzing the composition of T cells in the xenograft in more detail, a significantly increased proportion of CD4^+^ T cells was observed in the peripheral blood of NSG mice transplanted with cocultures of T cells and pDCs treated with both NGF and CpG A, compared to CpG A treatment only (*p* = 0.032; Figure 5E). On the other hand, the same coculture conditions resulted in a significantly lower number of CD8^+^ T cells (*p* = 0.047; Figure 5F). No significant influence of NGF on the proportion of CD4^+^ or CD8^+^ T cells in xenograft was detectable when transplanted pDCs were not stimulated with CpG A. Changes in the number of CD25^+^ T cells were not observed under the studied conditions.

In conclusion, GvHD triggered by pDCs presenting alloantigen and priming alloreactive T cells is regulated by p75NTR activity on pDC thus explaining the janiform role of pDC transplantation approaches described so far.

## Discussion

Plasmacytoid dendritic cells play a pivotal role in the modulation of immune responses. Here, we demonstrate, for the first time, that both human and murine pDCs express marked amounts of the low affinity neurotrophin receptor p75NTR, but not the high affinity neurotrophin receptors TrkA, TrkB, or TrkC (Figures 1A and 4A). Therefore, we studied the potential of neurotrophins to modulate the function of pDCs in immune responses.

The neurotrophin NGF is implicated in the regulation of inflammatory responses. In response to allergen stimulation, serum, and BALF from patients with allergic asthma exhibit highly increased levels of NGF correlating with the degree of airway hyperreactivity (17, 35). Intravenous injection of NGF into guinea pigs induces bronchoconstriction in response to inhaled histamine (36). Similarly, mice that overexpress NGF exhibit severe airway hyperreactivity in response to allergen exposure, while the administration of NGF-specific antibodies prevents allergen-induced bronchoconstriction (28, 37). Mutant Ex3 mice with systemically reduced p75NTR levels do not develop asthma in response to allergen challenge (38). Initially, the asthma-preventing effects induced by reduced p75NTR expression were attributed to cells of the nervous system. More recent reports suggest that the failure to develop asthma is caused by a reduced p75NTR expression by hematopoietic cells, although the immune effector cells have not been identified (39).

Therefore, we investigated the impact of p75NTR signaling in pDCs using a modified model of murine OVA-induced allergic asthma (29). We sensitized mice through the intratracheal application of OVA-loaded pDCs and induced an allergic reaction by subsequent exposure to OVA aerosol. Here, we demonstrate that OVA-loaded WT pDCs induce allergic asthma in mice, which was present with typical asthma-associated symptoms such as Goblet cell hyperplasia, perivascular inflammation, prominent eosinophilia, and lung inflammation (Figure 2). Further, the preincubation of WT pDCs with NGF strongly enhances the symptoms of allergic asthma (Figure 2).

To further investigate the role of p75NTR in pDC-induced asthma, we used Ex3 mutant mice. This mouse strain is deficient in the full-length transcript of p75NTR, but expresses an alternate isoform lacking exon 3 that encodes a receptor unable to bind neurotrophins (12). The surface marker profiles of primary pDCs derived from spleen and *ex vivo* differentiated BM-derived pDCs from Ex3 and WT mice were identical, indicating no defect in differentiation of pDCs in Ex3 mice (Figure 1A). We then applied, reciprocally, WT or Ex3 pDCs into each mouse strain, finding that the reduced susceptibility of Ex3 mice to developing allergic asthma was fully rescued by sensitization with OVA-loaded WT pDCs expressing physiological levels of p75NTR (Figure 2). By contrast, transplanting OVA-loaded Ex3 pDCs into WT mice failed to induce asthmatic reactions. We, therefore, provide first and direct evidence that effective sensitization of mice with OVA-loaded pDCs depends on functional p75NTR expression by pDCs.

Beside their role in the applied allergy model, pDCs have been described as a predictive marker for the outcome of allogeneic stem cell transplantation and the development of GvHD (8, 34). Therefore, we tested whether activation of p75NTR modulates the influence of pDCs on GvHD. Transplantation of primary human pDCs together with autologous pan T cells led to a profound GvHD in NSG mice. When the human xenograft was treated with NGF prior to the transplantation these mice deceased significantly earlier when transplanted cells were additionally stimulated by CpG A (Figures 5A,B). Interestingly, we observed a shift towards CD4^+^ T cells penalizing CD8^+^ T cells within the peripheral blood xenograft upon stimulation with NGF arguing for a selective T cell type dependent expansion (Figures 5C,D). Comparable alterations of the CD4/CD8 ratio have been observed during hematopoietic stem cell transplantation and development of GvHD both in clinic and mouse models (40, 41). But no connection to pDCs, p75NTR, or neurotrophins had been suggested.

To further illuminate the role of p75NTR signaling in pDCs, we performed *in vitro* experiments to study the effect of NGF on innate and adaptive immune responses. NGF treatment altered the production of cytokines in both murine and human pDCs. CpG A-induced IFNα production was abrogated by NGF treatment and this effect is specific for p75NTR since the addition of the p75NTR-selective, inhibitory TAT-fused peptide Pep5 to CpG-stimulated and NGF treated human pDCs restored the levels of IFNα (Figures 1B and 4C,D) (42). Conversely, the addition of NGF increased IL-6 production by WT pDCs stimulated with CpG B and human pDCs in response to IgE crosslinking (Figures 1B and 4G). This is consistent with the findings of Noga et al., who demonstrated increased IL-6 secretion by monocyte-derived human DCs in the presence of NGF and BDNF (43). Notably, Ex3 mice do not exhibit an exacerbated IFNα secretion as would be expected of a receptor mutant lacking the neurotrophin binding site. p75NTR signaling is complex and many, in part oppositional, effects are mediated by this receptor, e.g., cell survival and apoptosis depending on intracellular binding partners (9). There is evidence that p75NTR signal transduction does not operate in an on–off fashion but different signals seem to be transmitted by stimulated and unstimulated receptors, respectively (44). It was shown that neurotrophins bind to preformed and covalently linked p75NTR dimers which undergo conformational changes in a “scissors-like” manner after NGF binding (45). In addition, proteolytic cleavage of p75NTR by α- and γ-secretases was proposed as a signaling mechanism (46, 47). Since the Ex3 strain exhibits expression of a p75NTR isoform we suppose this isoform lacking the extracellular neurotrophin binding domain behaves as constantly active.

NGF treatment of murine and human pDCs revealed a functional influence of p75NTR signaling on antigen presentation and T cell priming (Figures 1E,F). In the presence of NGF-treated WT pDCs, CD4^+^ and CD8^+^ T cells showed opposite patterns of proliferation and cytokine secretion. This result is also comparable to our observations in the GvHD xenotransplantation model, which showed a bias toward CD4^+^ T cell proliferation in NSG mice transplanted with NGF-treated human pDCs (Figures 5E,F). Furthermore, the addition of NGF to cocultures of pDCs and CD4^+^ T cells increased the allergen-induced proliferation of T cells and the secretion of IL-5, a key factor for growth and eosinophil activation (Figures 4I,J). Since pDCs do not express high affinity neurotrophin receptors, the pDC-induced expansion of T cells and increased IL-5 secretion was likely due to regulation by the low affinity neurotrophin receptor p75NTR. Overall, these data indicate that NGF modulates cytokine secretion and T cell priming in a p75NTR-dependent manner, which has also been reported in human monocyte-derived dendritic cells and murine BM-derived conventional dendritic cells (48, 49).

To define the mechanisms by which p75NTR signaling regulates the function of murine pDCs, we analyzed the effects of NGF treatment and p75NTR deficiency on the TLR9 signaling pathway. CpG-stimulated murine WT pDCs responded with an alleviated TLR9 expression level in response to NGF in a concentration-dependent manner (Figure 1B). This was not the case for Ex3 pDCs, which generally expressed lower levels of TLR9 and IFNα in response to CpG (Figure 1B). TLR9 activation in pDCs results in the phosphorylation and nuclear translocation of transcription factors IRF3 and IRF7, which induce IFNα/β expression in response to viral infection and DNA sensing (50). We show here that TLR9-mediated phosphorylation of IRF3 and IRF7 is altered in cells treated with NGF (Figure 1C). More specifically, CpG A-induced phosphorylation of IRF3 and IRF7 is diminished by NGF treatment in WT pDCs, which provides a possible mechanism for the reduced IFNα production observed in NGF-treated pDCs stimulated with CpG A (Figures 1B and 4C). In addition, we detected lower levels of phosphorylation of IRF3, IKKα/β, and c-Jun in Ex3 pDCs, compared with WT pDCs, whereas the total amounts of these proteins were not altered. Using the neural crest-derived cell line PC12 Mamidipudi et al. and Wooten et al. demonstrated a direct, NGF-induced interaction between p75NTR, IRAK, the scaffold protein p62, IKKβ, and TRAF6 that activates NF-κB (51, 52). TRAF6, which is a critical factor for the maturation and T cell priming capacity of dendritic cells, interacts directly with p75NTR and regulates γ-secretase-mediated cleavage of p75NTR (47, 53). Notably, TRAF6 binds and activates IRF7 during TLR-induced IFNα production (54). Interestingly, the co-stimulatory molecules differentially expressed by OVA-stimulated WT and Ex3 pDCs are regulated by members of the family of IRF transcription factors (55, 56). It is noteworthy that in the absence of exogenously added NGF, WT pDCs expressing physiological levels of p75NTR displayed increased activation by CpG; higher levels of TLR9 and co-stimulatory molecules; increased phosphorylation of IRF3, IRF7, IKKα/β, and c-Jun; and a greater ability to stimulate T cells than Ex3 pDCs. These observations may be explained by p75NTR activation induced by NGF that is produced endogenously or by stimulated T cells, suggesting the presence of a p75NTR-dependent feedback loop between pDCs and other immune cells (57, 58).

Another promising, connective element of both TLR9 signaling, p75NTR and our observations in the different disease models is the small GTPase Rab5, which has been reported to form a complex with p75NTR in adipocytes (22). Rab5 plays a role in regulating the formation of endosomes, which are essential for initiation of TLR9-mediated signaling (59). It is, therefore, possible that p75NTR signaling regulates pDCs function by interacting with various molecules of the TLR9 signaling pathway.

TLR9 signaling contributes to various disease states, including the onset of autoimmune diabetes. In the NOD mouse, application of a TLR9 antagonist or depletion of type 1 IFN producing pDCs resulted in inhibition of diabetogenic CD8^+^ T cells and delayed diabetes onset (60, 61). Comparably, in an adapted murine model of autoimmune diabetes, we have shown that a transfer of antigen-loaded and CpG-stimulated pDCs treated with NGF led to an alleviated clinical phenotype in heterozygous RIP-CD80GP mice characterized by reduced glucose levels and delayed onset of diabetes compared with pDCs stimulated with CpG alone (Figure 3) (26). This aligns with the finding that NGF treatment of CpG-stimulated pDCs *in vitro* results in reduced expression of TLR9 and significantly lowers the level of IFNα. Collectively, our data suggest that p75NTR signaling plays a dual role in the regulation of pDCs function, since activation of this pathway abrogates pDCs functions that contribute to autoimmunity, but also promotes pDCs functions that enhance immune responses in models of OVA-induced asthma and transplantation-induced GvHD.

In conclusion, we found that pDCs express the low affinity neurotrophin receptor p75NTR and demonstrated that its expression reflects a basic phenotypic feature of pDCs. p75NTR mediates NGF-driven regulation of pDC-directed immune responses, including cytokine production and T cell priming. The results of functional experiments suggest that p75NTR-NGF signaling influences activation of IRF3, IRF7, c-Jun, and IKKα/β, revealing a novel regulatory circuit in pDC-mediated immune responses. Our central finding of functional expression of p75NTR by pDCs provides a promising target for future therapeutic interventions in allergic diseases, inflammatory disorders, and autoimmune diseases.

## Ethics Statement

All animal experiments were carried out in strict accordance with the German Animal Welfare Act. The protocol was approved by the Committee on Ethics of the Landesdirektion Dresden, Germany (Permit Number: 24-9168.11-1/2010-34, 24-9168.11-1/2013-32). Human cell material was used after donors granted informed consent with approval of the local ethics committee (EK 155052009).

## Author Contributions

JB, CR, MR, EB, MB, MA, CH, ST, and SB designed the experiments, interpreted the data, and wrote the manuscript. JB, MR, ST, KN, MB, MA, BD, and AJ performed the *in vitro* and *in vivo* studies. JB, MK, IR, DA, AD, and ST performed data analysis and statistical evaluation.

## Conflict of Interest Statement

The authors declare competing financial interests: CR, MR, SB, and ST filed a patent application describing the use of p75NTR to modulate the activity of plasmacytoid dendritic cells. All other authors declare that the research was conducted in the absence of any commercial or financial relationships that could be construed as a potential conflict of interest.
