# Supplementary figures and images for: Neurotrophin Receptor p75NTR Regulates Immune Function of Plasmacytoid Dendritic Cells
Source: Front Immunol. 2017 Aug 17;8:981. doi: 10.3389/fimmu.2017.00981 (PMC5562693; doi:10.3389/fimmu.2017.00981)

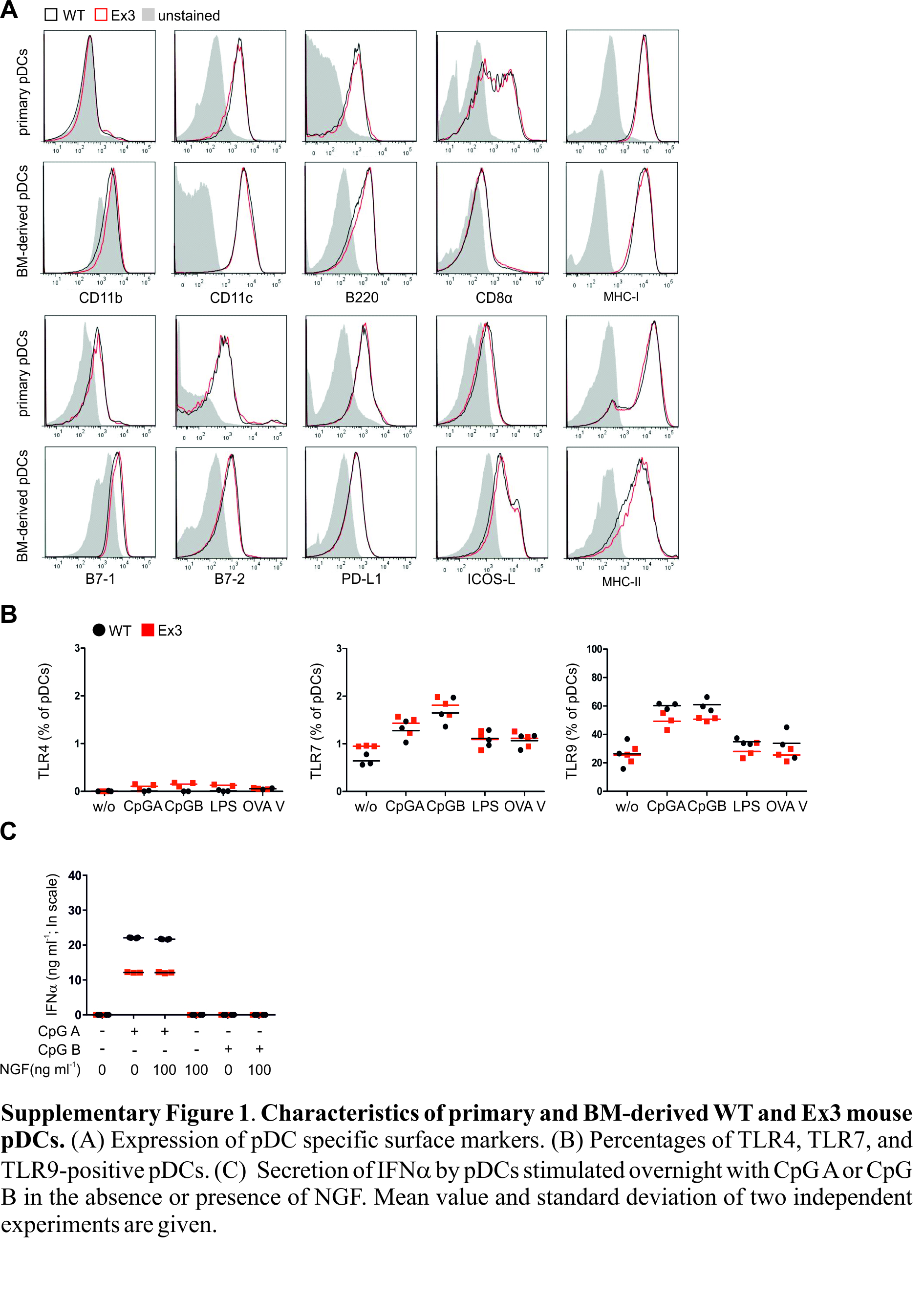

Supplement: Supplementary file 1 [file image_1.jpeg]

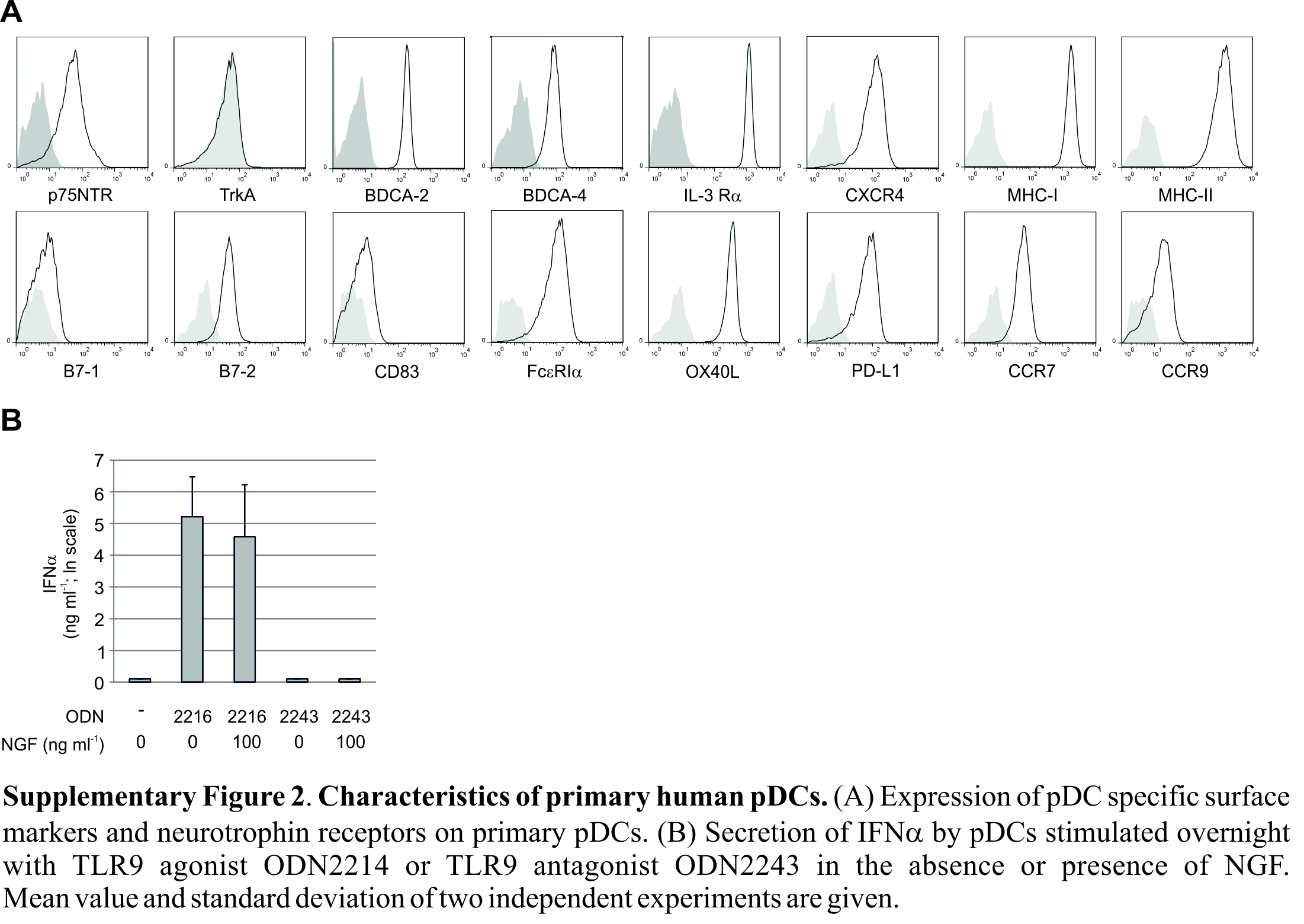

Supplement: Supplementary file 2 [file image_2.jpeg]
